# Decoding collagen cues: the interplay of integrins and discoidin domain receptors in health and disease

**DOI:** 10.1186/s12929-025-01211-0

**Published:** 2026-01-06

**Authors:** Paola Trono, Ilenia Masi, Flavia Ottavi, Laura Rosanò

**Affiliations:** 1 Institute of Biochemistry and Cell Biology (IBBC)-CNR, Via E. Ramarini, 32, 00015 Monterotondo Scalo, Rome, Italy; 2 Institute of Molecular Biology and Pathology (IBPM)-CNR, Via Degli Apuli 4, 00185 Rome, Italy

**Keywords:** Discoidin domain receptor (DDR) 1 and 2, Integrins, Cancer, Collagen, Targeted therapies, Aging, Fibrosis, Extracellular matrix (ECM), Tyrosine kinase receptor (TKR)

## Abstract

The extracellular matrix (ECM) provides critical biochemical and biophysical cues that regulate cell behavior in health and disease. Collagens dominate in abundance and structural importance, shaping tissue-specific ECM signatures that guide cellular behavior. Two major and distinct transmembrane receptor families, integrins and discoidin domain receptors (DDRs), serve as primary sensors for collagens, yet they employ fundamentally distinct binding mechanisms and signaling kinetics. While both can activate shared downstream pathways, their functional interplay remains complex and context-dependent, with the potential to fine-tune cellular responses to ECM cues. This review deciphers the nuanced crosstalk between integrin β1 and DDRs, with a particular focus on the understudied DDR2, across physiological and pathological processes. We discuss how this interplay, which evolves from cooperative to compensatory or even antagonistic signaling, is influenced by variables,  such as tissue specificity, developmental timing, and pathological context, dictating cell adhesion, migration, and ECM remodeling. Key examples include DDRs acting as allosteric regulators to license integrin activation, their partnership in mechanotransduction during development, and their divergent roles in aging tissues, where altered collagen mechanics shift the receptor hierarchy. In pathology, the DDR-integrin axis is pivotal in fibrosis and cancer, influencing fibroblast activation, drug resistance, metastatic outgrowth, and immune suppression within the tumor microenvironment. Notably, the receptors can function both independently and synergistically; for instance, DDR2 in cancer-associated fibroblasts regulates integrin-mediated mechanosignaling to promote metastasis, while in other contexts, both receptors activate distinct survival pathways. Understanding the signaling dynamics and mechanisms of these receptors is necessary for deciphering how cells interpret ECM signals and how these mechanisms contribute to disease progression, especially in those diseases marked by collagen remodeling. This comprehension is crucial for developing novel therapeutic strategies. Emerging evidence suggests that combined targeting DDRs and integrins can synergistically overcome ECM-mediated therapy resistance, enhance immune infiltration, and reprogram pathological microenvironments, offering a promising approach for treating fibrosis and collagen-rich cancers.

## Introduction

The extracellular matrix (ECM) represents the stage where the dynamic interplay of biochemical and biophysical signals between cells and their surrounding microenvironment occurs, regulating cell differentiation, function, and homeostasis [1]. These diverse functions are achieved through the ECM’s complex chemical composition and organization, in a way that the specific characteristics of each tissue result in a unique ECM signature that dictates tissue-specific cell behavior [2].

Among all the ECM components, collagens represent the most dominant, and their importance is highlighted by their contribution in the early evolutionary steps of multicellular animals [3]. Collagens are composed of three α chains characterized by repetitive G-X-X’ sequences, wherein the X position is often occupied by proline and X’ by 4-hydroxyproline [4]. The three α chains coil around each other to form a right-handed triple helical structure. Based on the ability of collagens to form highly ordered structures in tissues, they are categorized as fibril-forming collagens (types I–III), network-forming collagens (e.g., the basement membrane collagen type IV), and fibril-associated collagens with interruptions in their triple helices [5]. Collagen type I is the main component of the vertebrate ECM, making up ∼30% of the total protein mass in humans [6, 7]. It is abundant in the skin, tendons, blood vessels, and organs such as the lungs and heart. It also serves as the primary component of the organic matrix in calcified tissues as bone and teeth [8]*.*

Noteworthy, the integrity of the ECM deteriorates with age also due to accumulated damage from collagen fragmentation, oxidation, glycation, and crosslinking. This degradation compromises organ support and function, contributing to cellular aging and the progression of various diseases [9].

**Fig. 1:**
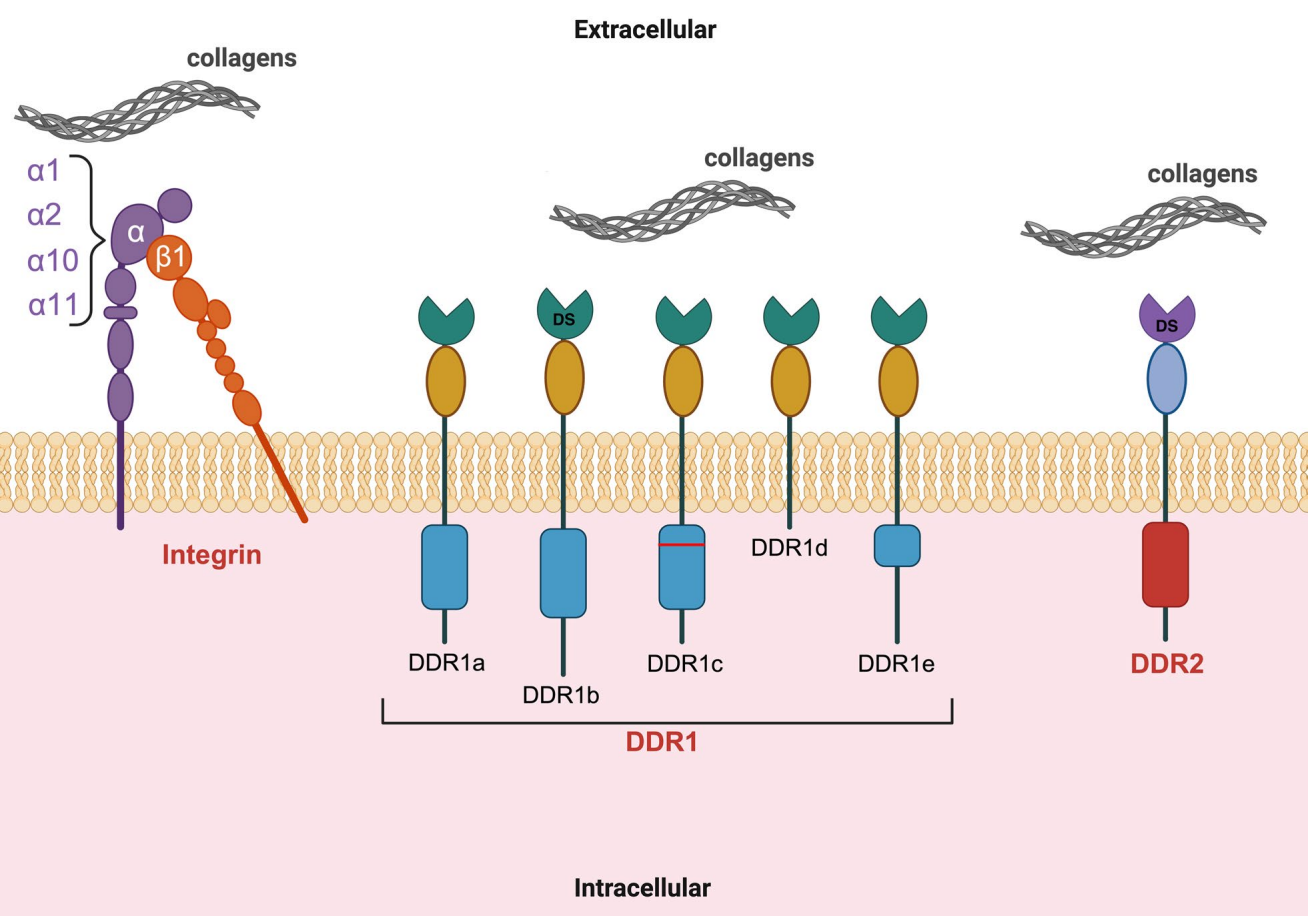
Intβ1, DDR1 and DDR2 schematic structure at the plasma membrane. Collagen-binding integrins are αβ1 heterodimers, with α1, α2, α10, and α11 serving as potential α subunits. The homologous discoidin domain receptors, DDR1 and DDR2, are characterized by the presence of the collagen-binding domain, namely discoidin homology (DS) domain, in their extracellular regions. Five isoforms of DDR1 have been identified

A structurally diverse group of transmembrane receptors allows collagen recognition. The emergence of collagen-binding integrins represents a pivotal milestone in the evolutionary history of vertebrate mechanobiology [10]. Another class of surface receptors, namely discoidin domain receptors (DDRs), first identified in the early 1990’s [11–13], has been found to bind collagens [14]. These two types of receptors not only recognize distinct regions of collagen fiber but also belong to distinct families with different structures and functions (Fig. 1).

**Fig. 2:**
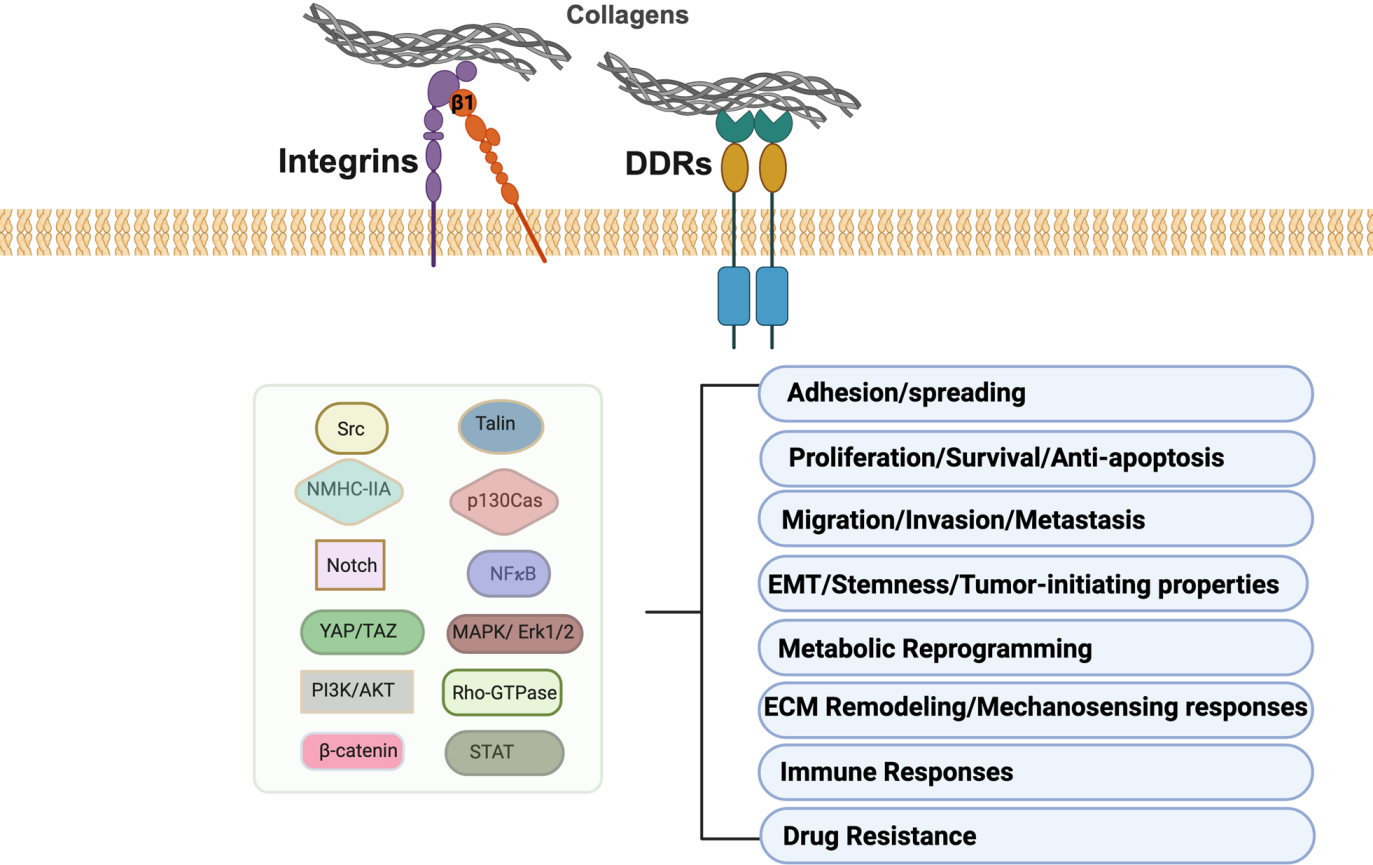
Overview of shared DDR and integrin β1 signaling. Schematic representation of DDR and integrin β1 interplay, depicting common key signaling intermediates and functional outcomes

Based on crystallographic analyses, DDRs and integrins engage in collagen through fundamentally distinct binding mechanisms. Indeed, despite both receptor families recognizing Glycine–Phenylalanine–Hydroxyproline (GFO) triplet motifs, they have evolved divergent strategies for collagen recognition, reflecting their unique structural and functional adaptations [15]. DDRs and integrins trigger shared downstream signaling pathways, such as Mitogen-Activated Protein Kinase (MAPK)/Extracellular Signal-Regulated Kinase (ERK), Phosphatidylinositol 3-Kinase/Protein Kinase B (PI3K/Akt), and Src kinase, that regulate key cellular processes (Fig. 2). However, each receptor type can also initiate distinct signaling mechanisms.

It is noteworthy that in a pioneering study it has been observed that full DDR1 activation after collagen stimulation also occurs in cells treated with blocking Abs for integrin (int) α2β1 or in cells with a targeted deletion of the intβ1 gene, demonstrating that DDR1 is activated independently of intβ1 [16]. Despite the paucity of studies reporting on the crosstalk between DDRs and integrins, it is highly probable that such crosstalk occurs. This is predicted by the fact that the activation of one influences or enhances the signaling pathways of the other, thereby enabling fine-tuned regulation of cell behavior in response to ECM cues. The collaborative, compensatory, or separate yet overlapping mechanisms through which these receptors operate are dictated by factors such as tissue type, developmental stage, and pathological condition. It is imperative to comprehend this interplay in greater depth to gain insights into how cells respond to their microenvironment and contribute to the progression of various diseases.

In this review, we will summarize evidence that sheds light on the complex and nuanced relationship between intβ1 and DDRs, with particular attention to the less-studied DDR2, across various physiological and pathological contexts. This remains a relatively underexplored area, despite its significant potential, especially in advanced diseases where collagen remodeling plays a pivotal role in driving poor prognosis. We will discuss the importance of integrating knowledge of the signaling mechanics and dynamics of these receptor families, aiming to support the development of novel therapeutic strategies capable of precisely modulating receptor-dependent cell behavior and signaling.

### Integrin β1: structure, activation, and signaling in collagen sensing

Integrins represent a major class among the ECM receptors for cell adhesion [17]. They are heterodimeric transmembrane glycoproteins composed of non-covalently associated α and β subunits, each with a large extracellular domain, a single transmembrane domain, and, typically, a short cytoplasmic domain. Among integrins, those able to recognize collagen belong to the intβ1 subfamily, central to the integrin family [18]. There are four collagen-binding integrins in mammalian cells: intα1β1, intα2β1, intα10β1, and intα11β1, able to bind specific amino acid motifs in the triple-helical regions. The major binding site in collagens I–III for intα1β1 and intα2β1 integrins is a GFOGER motif [19, 20]. Among all integrin-binding sites in collagen, the invariant residue is a glutamic acid, which coordinates the magnesium ion bound to the integrin I domain [21].

The conformational changes governing integrin-mediated adhesion and signaling are intricate, involving multiple structural states associated with either low or high affinity for extracellular ligands [17, 22]. In their inactive form, as observed in adherent cell types such as fibroblasts, epithelial cells, and cancer cells, integrins typically adopt a compact, bent conformation, with the ligand-binding headpiece oriented toward the plasma membrane and the cytoplasmic "legs" bound by cytoplasmic integrin inhibitors. Integrin activation can be induced by both “inside-out” and “outside-in” signals. The "inside-out" signals are triggered by intracellular signaling, whilst the "outside-in" signals are prompted by extracellular ligand binding [23]. The biochemical activation of the “inside-out’ signaling promotes the assembly of a large multiprotein assembly known as the integrin adhesion complex (IAC), mediated by scaffold proteins like talin or kindlins, which links integrins to the actin cytoskeleton and other adhesome components. Upon activation, integrins transit to a fully extended conformation, projecting the headpiece outward toward the extracellular environment, enhancing ligand binding and triggering downstream signaling events. Through this complex, integrins transduce ECM signals into the cytoplasm, despite lacking intrinsic enzymatic activity, through focal adhesion kinase (FAK) and Src-mediated phosphorylation of IAC proteins and cytoskeletal components. Furthermore, activation of integrin-related pathways, such as FAK, Src, AKT, ERK, and RHO GTPase family, can be triggered by engagement of integrin with ECM ligands and their subsequent clustering and accumulation of IAC complexes via “outside-in” integrin signaling [5, 24, 25]. The dynamic and complex integrin activity is regulated by biochemical factors, scaffold proteins, ECM ligands, cations, and mechanical forces, and cells fine-tune responses to ECM signals and associated processes through this integrin bidirectional signaling.

### DDR1 and DDR2: structure, binding kinetics, and signaling

Differently from integrins, DDR1 and DDR2 are receptor tyrosine kinases characterized by the presence of a discoidin homology (DS) domain in their extracellular regions containing a collagen binding site [14, 26]. DDRs display unique structural features, with six distinct protein domains: the DS domain, a DS-like domain, an extracellular juxtamembrane region, a transmembrane segment, a cytoplasmic juxtamembrane region, and a C-terminal kinase domain, where the phosphorylation of target proteins triggers downstream signaling cascades critical for DDR-mediated cellular responses [5]. Upon binding primarily fibrillar collagens, rather than by peptide growth factors, DDRs undergo tyrosine autophosphorylation, like other receptor tyrosine kinases (RTKs), but, differently from them, the activation process is characterized by unusually slow and distinct kinetics [14, 26], triggering a range of cellular programs, including cell adhesion and mechano-transduction processes, whose molecular mechanisms are still not fully understood. A key aspect of the DDR2-collagen interaction, accurately predicted through structural modeling [27], is that the critical collagen residues involved in binding originate from different collagen chains. The apolar GVMGFO motifs of two collagen chains are recognized by an amphiphilic pocket in DDR2, in a manner that is fundamentally different from the metal ion-dependent mechanism employed by integrins [15, 28]. This requirement explains why a triple-helical collagen structure is essential for DDR2 binding [29]. Ligand binding promotes Src to phosphorylate tyrosine residues in the DDR2 activation loop (Tyr-736, Tyr-740, and Tyr-741), stimulating intramolecular autophosphorylation of additional tyrosine residues [30, 31]. Specifically, Type I collagen (Col1) binding promotes Src-dependent phosphorylation of Tyr-740, which in turn stimulates DDR2 cis-autophosphorylation of additional tyrosine residues, demonstrating that the catalytic activity of DDR2 and Src promotes DDR2 tyrosine phosphorylation [30, 31]. In addition, DDR2 autophosphorylation fosters the formation of DDR2 cytosolic domain-Shc signaling complexes. Similarly, Src-mediated phosphorylation of DDR1 is required for full activation of DDR1 [32, 33]. Thus, DDR-Src interactions are crucial in initiating DDR signaling [34]. While adaptor molecules, including ShcA [26, 30] and Nck2 [35] have been reported to be recruited at DDR1, limited knowledge of intracellular signaling partners for DDR2 exists, and downstream DDR2 activation is still in part unknown [34]. It is noteworthy that significant, well-established signaling pathways, including MAPK and Akt, undergo regulation by DDRs. However, the comprehensive characterization of signaling pathways downstream of DDRs remains to be fully elucidated.

As collagen reorganization is central to shaping tissue mechanics in diverse cellular processes driving development, tissue repair, and pathological states [36, 37], DDR2, as major transducer of collagen cues, senses collagen dynamicity and activates intracellular pathways, with a profound impact on cell behaviors. Strikingly, DDR2-driven signaling pathways active in physiological contexts are expected to mediate more than those known so far also in pathological contexts like cancer.

During embryonic development, collagen organization is tightly coordinated with morphogen gradients and cell differentiation. Initially, collagen fibers are loosely organized to permit high plasticity for cell migration and organ shaping; as tissues mature, fibers progressively align and undergo cross-linking. DDR2 plays an essential role in these processes, as demonstrated in bone development [38], where it is required for proper collagen organization, chondrocyte proliferation, and orientation [39]. Consistently, Ddr2 loss-of-function mutations in humans and mice cause severe skeletal defects [40, 41]. Recent evidence further implies DDR2 as a modulator of bone morphogenetic protein (BMP) signaling through regulation of Hippo pathway responses to collagen matrices [42]. Beyond bone, DDR2 also influences cardiac lineage specification by controlling cell–matrix adhesion and BMP-Smad signaling [43]. Notably, DDR1 has been implicated in a signaling pathway that drives breast epithelial differentiation in cooperation with RUNX1, a key regulator of epithelial–mesenchymal transition (EMT) in breast cancer [44]. The DDR1–RUNX1 axis functions as a critical transcriptional node, orchestrating stem cell signaling, epithelial differentiation, and shaping the morphological architecture of breast tissue. Dysregulation of this pathway may contribute to breast cancer pathogenesis [45].

Collagen reorganization is a fundamental process during tissue repair following injury, essential for restoring tensile strength, where the reactivation of the cellular and molecular programs used during embryonic development is needed. The involvement of DDR2 in this context is deeply reviewed in [46]. An illustrative example is DDR2’s role in skin fibroblast responses following tissue injury, where DDR2 orchestrates collagen processing and contributes to the restoration of wound tensile strength [47]. DDR2 phosphorylation initiates key wound-healing responses in fibroblasts, including proliferation, chemotactic migration, and secretion of both matrix metalloproteinases (MMPs) and fibrillar collagen. The pivotal role of DDR2 in tissue repair has prompted investigations into the development of advanced materials, also considering integrin role in tissue repair, as therapeutic targeting applications in regenerative medicine, as discussed later [42, 48].

Strikingly, DDR2 ability to interpret collagen cues is co-opted by tumor cells that exploit DDR2 signaling to activate pathways (e.g., MAPK and Hippo pathway) to sustain invasion and metastasis.

Collagen synthesis and remodeling dominate the desmoplastic response characteristic of many tumors, where fibers become densely packed, highly cross-linked, and aligned, dramatically increasing tissue stiffness and altering mechanotransduction [49]. DDR2 is aberrantly expressed in numerous advanced malignancies and is strictly involved in collagen deregulation-driven processes that promote invasion and metastasis, including EMT programs [50]. Multiple evidence connects DDR2 and EMT to tumor aggressiveness across cancer types, as in metastatic melanoma, where it sustains the EMT-like process of phenotype switching and MAPK pathway activation and proliferation in melanoma BRAF-resistant cells [51], or in gastric cancer where DDR2 promotes stemness maintenance and EMT of gastric cancer stem cells by mTOR-SOX2 axis [52]. In metastatic triple-negative breast cancer (TNBC), DDR2 is highly expressed along with the EMT inducer MMP14 [53]. DDR2 is highly expressed in recurrent breast tumor cells displaying EMT features and promotes ferroptosis by activating YAP/TAZ [54]. Noteworthy, different recent studies have proposed DDR inhibition as a promising strategy to overcome cancer therapy resistance in a range of aggressive cancer, based on the promotion of ferroptosis, an iron-dependent programmed cell death modality driven by lipid peroxide accumulation [55]. Hu et al. has highlighted the potential of DDR1 inhibitors as radiosensitizing agents in head and neck squamous cell carcinoma, where DDR1 overexpression is linked to immune evasion and poor prognosis [56]. Mechanistically, DDR1 forms a ternary complex with 14–3-3 and Akt, which drives monounsaturated fatty acid synthesis and inhibits ferroptosis. Both genetic and pharmacological inhibition of DDR1 disrupts this complex, enhancing ferroptosis, increasing tumor immunogenicity, and promoting CD8^+^ T cell infiltration, with the amplification of the antitumor efficacy of carbon ion radiotherapy [56]. DDR1 inhibition promotes ferroptosis and restores gefitinib sensitivity in non-small cell lung cancer (NSCLC) [57]. Gefitinib-resistant NSCLC cell lines exhibit elevated DDR1 expression, which regulates the miR-3648–SOCS2 axis, with SOCS2 acting as a positive modulator of ferroptosis. Blocking this axis indirectly enhances ferroptosis through the SLC7A11–GPX4 pathway [57].

DDR2 can confer protection against ferroptosis in ovarian tumor-associated fibroblasts (CAFs) by regulating the xCT–GSH–GPX4 antioxidant pathway and cellular iron metabolism [58]. Moreover, CAFs secrete DDR2-dependent factors that shield ovarian cancer cells from cytotoxicity induced by olaparib-, a clinically relevant PARP inhibitor (PARPi). Thus, targeting stromal DDR2 may enhance ferroptosis sensitivity and improve ovarian tumor responses to PARPi therapy [58].

Overall, the outcomes of DDR-induced signaling pathways have been observed to vary, exhibiting context- and cell-type-dependent characteristics [34]. This observation underscores the necessity for more comprehensive studies to elucidate the intricacies of these signaling processes, in both space and time.

### Functional interplay between intβ1 and DDRs

While it seems intuitive that intβ1 and DDRs might interact, given their shared ligands (collagens) and overlapping biological roles, evidence of a direct crosstalk between them is still scarce. As deeply reported below, key studies have investigated the interaction of these receptors across diverse cell types, including epithelial and mesenchymal cells, most of them focused on fibroblasts. This preference is justified considering that fibroblasts are the primary ECM-producing and -remodeling cells, playing central roles in fibrotic diseases and cancer stroma [59]. During tumor progression, they differentiate into CAFs, acquiring pro-tumorigenic functions and becoming critical regulators of the tumor microenvironment (TME) [59]. Fibroblasts express exceptionally high levels of DDR2, making them a predominant model for studying DDR-mediated collagen sensing and adhesion. Moreover, in fibroblasts the intβ1 subunit serves as a key mediator of mechanotransduction, essential for tissue homeostasis, repair, and fibrosis [60]. In cancer, intβ1 promotes tumor invasion, with CAF-driven ECM remodeling further dysregulating integrin signaling pathways [61]. As the ECM undergoes dynamic changes in composition and mechanical properties in these pathological contexts, the functional interplay between intβ1 and DDRs emerge as a critical regulatory node that needs to be deeply characterized. Growing interest in this field is expected to yield new insights into these interactions in the near future.

**Fig. 3:**
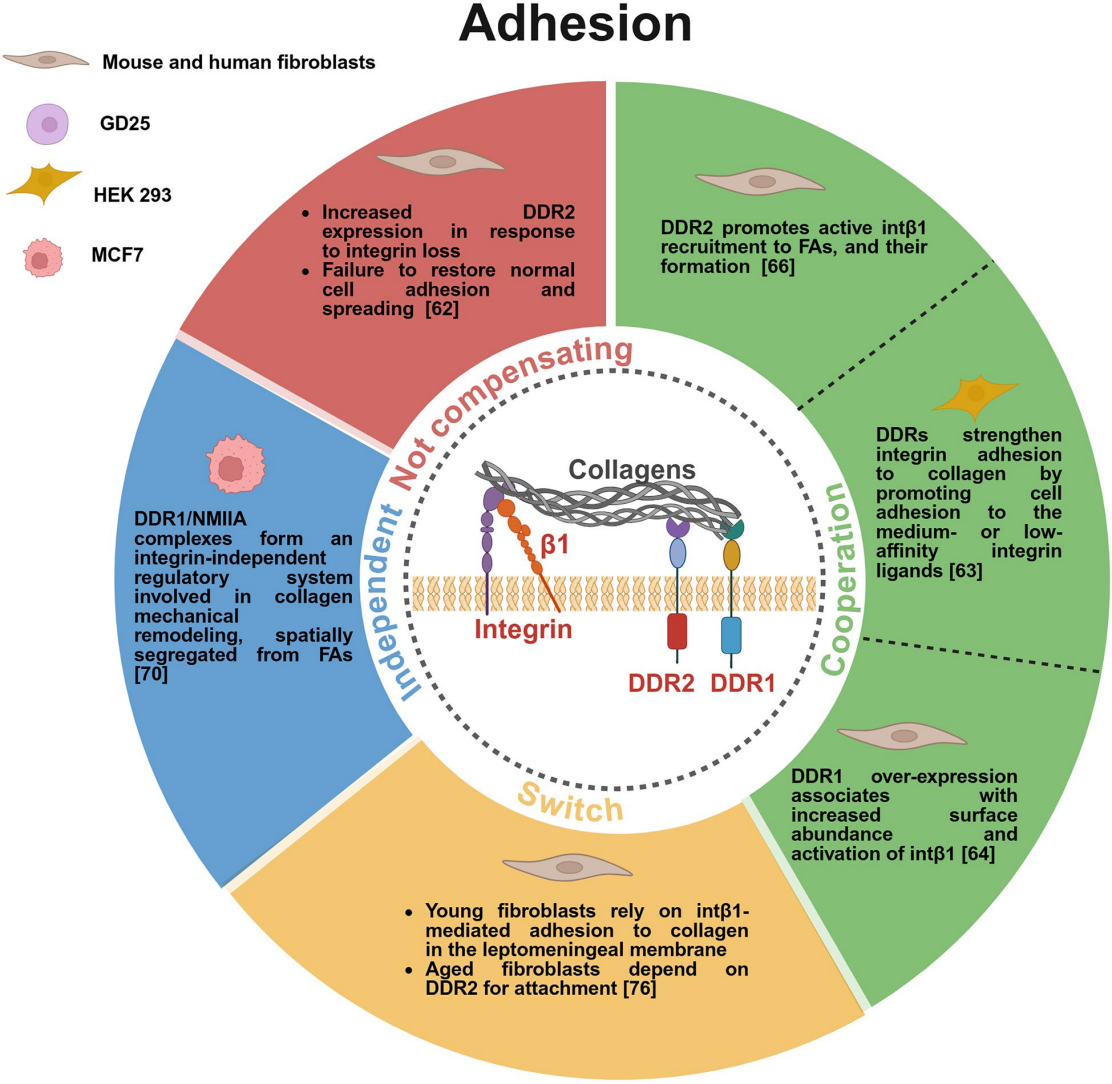
DDRs and intβ1 crosstalk in cell adhesion. Graphical representation of the different modes of interactions between DDRs and intβ1 in different cellular systems (green for cooperation, yellow for switch, blue for independent, red for not compensating)

**Fig. 4:**
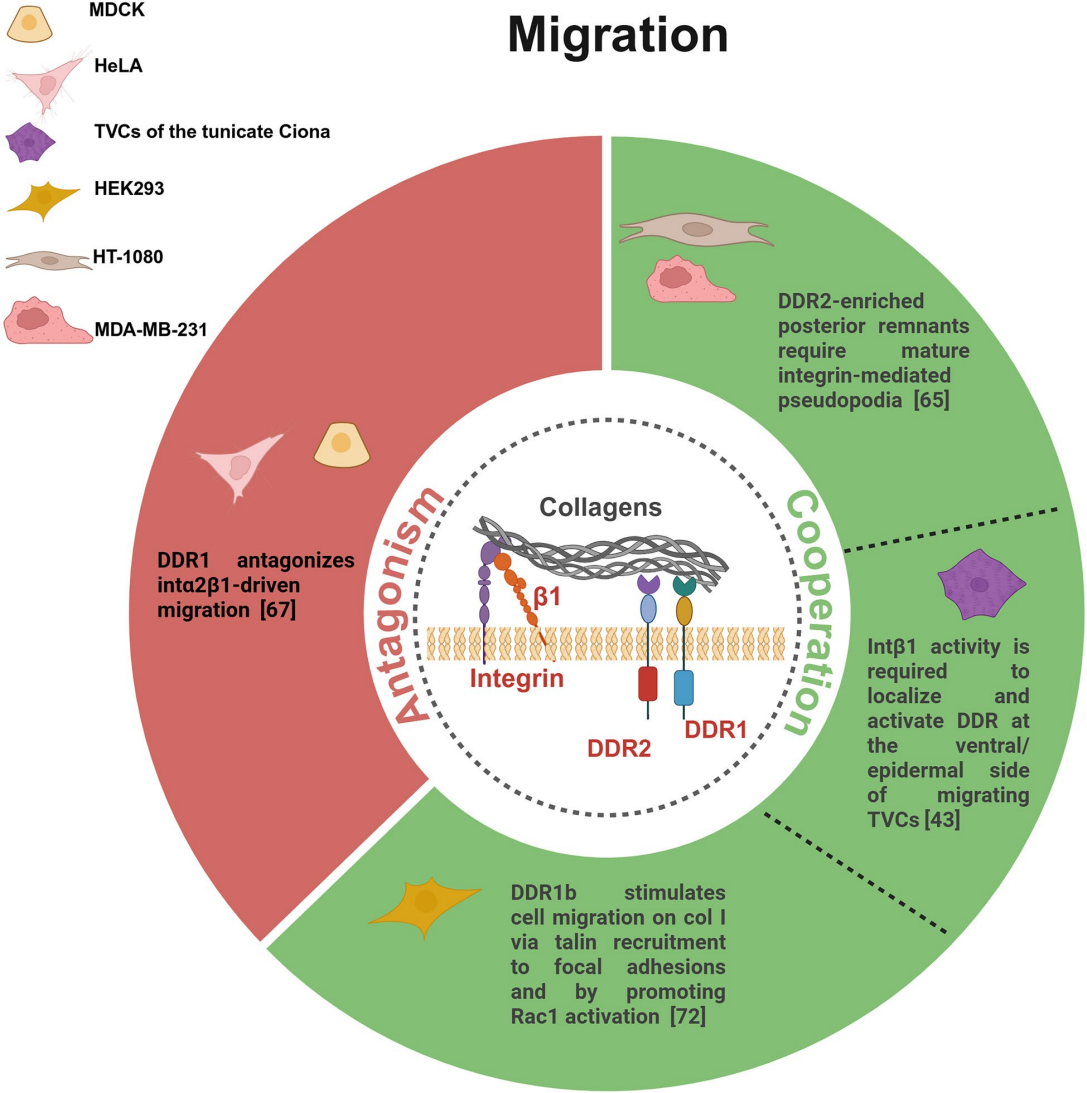
DDRs and intβ1 crosstalk in cell migration. Schematic illustration depicting the opposite interaction modes between DDRs and intβ1 in different cellular contexts (green for cooperation, red for antagonism)

Herein, we report key findings demonstrating a functional interplay between intβ1 and DDRs. This interaction results in context-dependent outcomes under both physiological and pathological conditions (Figs. 3 and 4).

### Intβ1 and DDRs crosstalk in relevant physiological processes: adhesion and migration

To investigate integrin-mediated cell-collagen communication, triple knockout mice have been generated with a combined ablation of intα1β1, intα2β1, and int α11β1 [62]. Analysis of the adhesive behavior of primary dermal fibroblasts derived from these mice revealed that the absence of these integrins leads to a compensatory upregulation of DDR2. Despite the elevated levels of DDR2, fibroblasts exhibited severely impaired mechanotransduction, reduced collagen deposition, and disorganized matrix structure. Importantly, DDR2 was unable to compensate for the loss of integrin function, as adhesion and cell spreading remained defective (Fig. 3). This demonstrates that DDRs cannot substitute for integrins as primary adhesion receptors in dermal fibroblasts, despite their shared collagen-binding capacity [62].

Consistently, neither DDR1 nor DDR2 supports firm adhesion to collagen themselves, but they regulate integrin-dependent mechanisms, as elegantly demonstrated in a study of the Leitinger group, where the individual contributions of DDRs and intβ1 to cell adhesion were addressed by employing receptor-selective synthetic triple helical collagen-like peptides [63]. The authors demonstrated that DDRs strengthen integrin adhesion to collagen by promoting cell adhesion to medium- or low-affinity integrin ligands in HEK293 cells (Fig. 3). Specifically, DDRs regulate the activation state rather than the expression level of the integrins, likely leading to a more active conformation. Based on that, the authors define DDRs as not true adhesion receptors and suggest that the contribution of the DDRs may be more prominent in tissues where high-affinity integrin binding sites are either not exposed on collagen or inaccessible, acting as an allosteric regulator [63]. From a functional point of view, DDR-dependent integrin “licensing” could enable cells to sense and, when possible, remodel ECM also in refractory niches. How exactly do DDRs conformationally activate integrins remain to be established.

Notably, it has been demonstrated that DDR1 over-expression in mouse and human fibroblasts associates with increased surface abundance and activation of intβ1. This effect appears to be mediated, at least in part, by DDR1-regulated post-translational modifications, implicating DDR1 in post-translational control of integrin trafficking or conformational priming of intβ1 (Fig. 3) [64].

While these studies highlighted that integrins act as mechanical anchors and DDRs as signaling amplifiers that optimize integrin responsiveness to collagen, other pivotal studies demonstrated their partnership through receptor cooperativity. A critical collaboration between the two collagen receptors comes from studies on a mechanism known as posterior remnant tethering. During the retracting phase of migrating cells, DDR2 is recruited to the distal end of retracting pseudopodium, where it binds collagen fibers and forms stable clusters within the matrix [65]. Following de novo formation, these DDR2-enriched posterior remnants are incorporated into the collagen matrix through an integrin-dependent process, which requires the maturation of integrin-mediated pseudopodia. Interestingly, Intβ1 is not present in these posterior remnants at the rear of a retracting pseudopodium, indicating spatial segregation between DDR2 and integrin adhesion domains (Fig. 4). Consistently, DDR2-based posterior clusters and integrin-based focal adhesions appear as distinct cell adhesive domains. Importantly, the loss of integrin function is not rescued by DDR2, underscoring their distinct roles in cell adhesion and migration [65]. Notably, DDR2 collagen binding activity, rather than its kinase activity, is crucial for the formation of posterior remnant.

In the context of developmental process, a collaboration between Ddr and Intβ1 promotes cell–matrix adhesion of the cardiac lineage in the tunicate Ciona [43]. During embryogenesis, cardiopharyngeal progenitor cells (namely trunk ventral cells, TVCs) collectively polarize and migrate between the ventral epidermis and trunk endoderm. Before the onset of migration, the trunk endoderm secretes type IX collagen that activates Ddr on the ventral surface of TVCs, thereby promoting integrin-based adhesion to the underlying epidermis. Of note, Intβ1 activity is required to localize and activate Ddr at the ventral/epidermal side of migrating TVCs. Furthermore, the frequency of adhesion defects in TVCs co-expressing dominant-negative constructs of Ddr and intβ1 is not significantly higher than in single perturbations, suggesting that Ddr and intβ1 act in overlapping pathways to regulate cell–matrix adhesion (Fig. 4). Interestingly, Ddr-but not intβ1-also promotes BMP-Smad signaling, which is essential for establishing and maintaining TVC polarity. This indicates that in this developmental context, Ddr supports both cell adhesion and polarity via distinct, non-redundant signaling mechanisms [43].

A close collaboration between DDR2 and intβ1 has also been reported in studies on the alveolar formation in the lungs of mice carrying DDR2 deletion [66]. Impaired cell spreading, reduced lamellipodia formation and blunted responsiveness to collagen rigidity were observed in DDR2-deleted fibroblasts, associated with reduction in ventral stress fibers and phospho-zyxin-enriched focal adhesions, where less activated intβ1 is evident (Fig. 3). This study has revealed that DDR2 directs the recruitment of active intβ1 to focal adhesions in response to substrate stiffness, implying that DDR2 primes cells to interpret mechanical cues from the developing lung ECM, ensuring the correct linear focal adhesion alignment, a hallmark of polarized force transmission critical for tissue morphogenesis [66]. Noteworthy, another study has highlighted an opposite signaling between DDR1 and intα2β1 during hepatocyte growth factor (HGF)-induced branching morphogenesis, where DDR1 acts as a negative regulator of integrin-driven migratory responses [67]. While intα2β1-mediated signaling modulates collagen-induced cell migration by regulating tyrosine phosphorylation of Stat1 and Stat3, DDR1 counteracts this signaling by recruiting the phosphatase SHP-2, which dephosphorylates Stat1/Stat3 without altering intα2β1 expression levels (Fig. 4) [67]. The balance between DDR1 and intα2β1 may ensure that migration occurs only in specific niches, where DDR1’s restraint could prevent excessive or misdirected cell movement. Such reciprocal regulation likely extends to other collagen-rich contexts, with therapeutic potential in diseases of dysregulated motility. It is interesting to note that SHP-2 recruitment by DDR1 has also been shown to inhibit intβ1-Src signaling in a study where DDR1 was found to stabilize E-cadherin. While intβ1 promotes Src activation and E-cadherin endocytosis, DDR1 counteracts this process by recruiting SHP-2 [68]. Thus, DDR-integrin crosstalk functions as a context-sensitive regulator of adhesion and signaling, integrating cues from ECM architecture and cellular phenotype to determine epithelial stability or motility.

The traditional view of DDRs as ancillary collagen receptors working in concert with intβ1 has been fundamentally revised by recent studies demonstrating their capacity for autonomous matrix sensing and cell-matrix interaction, thus revealing a previously underappreciated role of DDRs in cell adhesion and migration. DDR1 is known to be associated with non-muscle myosin IIA (NMIIA) [69]. By employing integrin null cells, Coelho et al. demonstrated that DDR1/NMIIA adhesion complexes are involved in collagen mechanical remodeling, and are spatially segregated from focal adhesions, forming an integrin-independent regulatory system (Fig. 3) [70]. This mechanism may be critical in contexts where integrins are downregulated. A specific intβ1-independent DDR1 role has also been suggested in leukocytes migration through the ECM, as the adhesion on collagen of DDR1a overexpressing THP-1, a human monocytic leukemic cell line, is not inhibited by anti-intβ1 blocking Abs (Table 1) [71]. This underscores a specialized role for DDR1 in immune cell trafficking and could explain how immune cells may navigate dense collagenous structures even when integrin functions are compromised.

**Table 1:** Different modes of interaction between DDRs and intβ1 in cancer

Biological process	Experimental model	Mode of interaction	Key findings	Citations
Adhesion	Breast tumor CAFs	Cooperation	DDR2 controls full collagen-binding intβ1 activation via Rap1-mediated Talin1 and Kindlin2 recruitment	[82]
Adhesion	BRAF-mutant melanoma cells	Cooperation only when interacting	DDR1 and intβ1 interaction is regulated by Kindlin-3 sustaining cell adhesion	[86]
Migration	HT-1080 fibrosarcoma cells, MDA-MB-231 human breast cancer cells	Cooperation	During retraction, DDR2-enriched posterior remnants are incorporated into the collagen matrix, requiring mature integrin-mediated pseudopodia	[65]
Migration	THP-1 human monocytic leukemic cell line	DDR1 specific, intβ1-independent	DDR1a-dependent cell adhesion is not inhibited by anti-intβ1 Abs	[71]
Matrix-mediated drug resistance (MMDR)	Fibroblast-derived 3D ECMs and melanoma cells	Specific to DDRs, not shared	BRAF inhibitors induce linear clustering of pDDR1/DDR2; MMDR is not reversed by intβ1 blockade or FAK depletion	[83]
Cell fate	Breast tumor cells	Alternative with opposite outcome	DDR2 signaling promotes tumor cell quiescence; integrin signaling enhances stem-like traits and tumorigenic potential	[93]
Proliferation	Pancreatic cancer cells	DDR1 downstream to intβ1	Col1 homotrimer-intα3β1 signaling induces DDR1 activation along with proliferative signals; DDR1 inhibition does not abrogate proliferative signals	[95]
Survival	Ovarian cancer cells	Compensating	In the absence of ITGB1, DDR1 sustains matrix-induced resistance	[87]
Survival	Breast cancer cells	Compensating	In the absence of ITGB1, DDR1 acts as a primary collagen sensor and maintains MAPK activation	[89]

A list of evidence has highlighted distinct interaction types between the two receptors demonstrating their functional effects across multiple biological processes in cancer. The specific experimental models used are provided

A dual role of DDR1b as an intβ1 collaborator and an autonomous motility driver has been identified from Borza et al. in HEK293 cells. Indeed, DDR1b stimulates cell migration on collagen I not only cooperating with intβ1 via talin recruitment to focal adhesions, but also directly by promoting the activation of the small GTPase Rac1 [72].

This functional divergence suggests that collagen sensing occurs through multiple parallel systems, with DDRs providing specialized capabilities that complement rather than mimic integrin functions.

### DDR2 and intβ1 crosstalk in adhesion processes modifies with aging

Interestingly, chronological aging and age-related pathologies are characterized by progressive collagen modifications particularly through non-enzymatic glycation, leading to with advanced glycation end-products (AGEs) formation [73]. These biochemical modifications are accompanied by increased cross-linking of collagen fibers and significant alteration in the structural and mechanical properties of the fibrillar type I collagen, leading to characteristics increases in tissues stiffness and decreased matrix turnover, hallmarks of aged and dysfunctional matrix [74]. While Saby et al. investigated how collagen aging affects the proliferation of human fibrosarcoma HT-1080 cells, they observed striking differences in DDR2 activation depending on the collagen source [75]. Cells cultured on adult rat collagen showed robust DDR2 activation, whereas those grown on aged rat collagen exhibited reduced DDR2 signaling accompanied by enhanced proliferation. Interestingly, intβ1 inhibition does not affect cell proliferation, thus excluding its involvement in this context [75].

Parallel studies in meningeal fibroblasts demonstrated that age-dependent DDR2 and intβ1 expression levels change with aging. In aged mice, the brain meningeal membrane exhibits structural and mechanical alterations characterized by accumulation of AGEs, discontinuous distribution of collagen and loss of tissue organization. Interestingly, meningeal fibroblasts adhesion mechanisms switch with age. While young fibroblasts rely on intβ1-mediated adhesion of collagen in leptomeningeal membrane, aged fibroblasts increasingly depend on DDR2 of the attachment [76]. This shift correlated with upregulated DDR2 expression and downregulated intβ1 level, altering mechanosensitivity [76]. These studies collectively reveal an ECM-age-dependent receptor hierarchy where collagen modifications dictate whether DDR2 or intβ1 dominates cell-ECM crosstalk, with implication not only in aging and related pathologies, but also in cancer and fibrotic disorders.

### Intβ1 and DDRs crosstalk in pathological processes: fibrosis and cancer

In physiological conditions, fibrillar collagen remodeling occurs primarily by tightly regulated pericellular focal proteolysis and phagocytosis. In contrast, in pathological states, such as fibrosis or throughout cancer progression, this homeostatic balance is disrupted, leading to alteration in the biomechanical properties of ECM [1, 2]. Characteristic features include aberrant collagen remodeling patterns by enhanced alignment, compaction, and stiffness of fibrillar collagen arrays. These changes create a self-reinforcing cycle of ECM disfunction driving disease progression [77]. Integrins and DDRs acquire a critical role especially in these pathological contexts characterized by the abundance of their shared ligand, Col1. In the injured myocardium, Col1 deposition during tissue remodeling is mediated by cardiac fibroblasts, which serve as the unique intracardiac source of this fibrillar collagen. This process is primarily mediated by Angiotensin II (Ang II) signaling through the AT1 receptor, where elevated levels of Ang II act as potent pro-fibrotic stimulus following injury [78]. Kailasam’s group has demonstrated that the DDR2-integrin crosstalk integrates ECM signaling and Ang II-mediated fibrotic pathways in cardiac fibroblasts. Specifically, Ang II induces the expression of DDR2 which, in turn, regulates intβ1 expression via ERK1/2-dependent transforming growth factor-1 (TGF-β1) activation. Of note, intβ1 regulates α-SMA and Col1 expression. These findings establish a reciprocal regulatory loop where intβ1 places downstream of DDR2 and link DDR2 to α-SMA, Col1, and wound healing. The link between DDR2 and intβ1 is confirmed in two genetic models: in spontaneously hypertensive rats, intβ1 expression positively correlates with DDR2 expression, and DDR2-null mice exhibit significantly reduced myocardial intβ1 [79], confirming DDR2 upstream regulatory role.

Later, it has been demonstrated that DDR2 mediates Ang II-stimulated fibronectin expression via YAP, a process critical for cardiac fibroblast survival under oxidative stress conditions. This establishes a feed-forward mechanism where DDR2-dependent fibronectin production activates intβ1/Integrin-Linked Kinase signaling, that in turn upregulates the expression of AT1R, further potentiating fibrotic responses. Based on this evidence, DDR2 has emerged as a cardiac fibroblast-selective drug target to control cardiac fibrosis [80].

ECM, and particularly Col1, acquires peculiar importance in cancer by shaping TME architecture, influencing tumor development, and response to therapy [81]. Although both DDRs and integrins serve as key sensors of these changes, their functions and the interplay between them vary across cancer types, reflecting context-dependent signaling crosstalk and therapeutic vulnerabilities (Table 1). In breast cancer, DDR2 acts as regulator of integrin-mediated mechano-transduction within the primary tumor. In murine models of breast tumor, Longmore’s group demonstrated that DDR2, primarily expressed in CAFs, reorganizes collagen fibers at the tumor-stromal boundary, increasing stiffness and promoting metastasis, by generating a physically permissive TME [82]. Mechanistically, DDR2 controls Talin1 and Kindlin2 recruitment to integrin adhesion complexes, enabling full activation of intβ1 collagen binding at cell protrusions. Thus, defective mechano-biologic properties observed in DDR2 lacking mice, including altered collagen fiber organization and decreased tumor stiffness, are likely due to defective collagen binding intβ1 activity [82].

Divergent roles of DDRs have been observed in drug resistance. In melanoma, a non-cooperative DDR2/intβ1 axis drives matrix-mediated drug resistance [83]. Fibroblast-derived ECM protects melanoma cells from BRAF^V600^-targeted therapies by inducing DDR1 and DDR2 linear clustering. Pharmacological inhibition of DDR1/2 effectively abolished ECM-mediated resistance and prevented collagen fiber area and thickness, independently of intβ1/FAK/MAPK signaling, contrarily to that demonstrated by previous studies implicating intβ1 in adaptive resistance through fibronectin-mediated FAK and MAPK pathway reactivation [84, 85]. Instead, DDRs activate a distinct NF-κB2/RelB pathway, potentially regulated by increased actomyosin contractility.

In BRAF-mutant melanoma cells, an interaction between DDR1 and intβ1, regulated by Kindlin-3, has been observed. Kindlin-3 is a tumor suppressor protein involved in focal adhesion, whose expression in melanoma patients is associated with better survival and DDR1 expression. Loss of Kindlin-3 disrupts DDR1/intβ1 interactions and leads to reduced adhesion and enhanced tumor progression [86]. These findings suggest a further level of regulation acting to inhibit tumor progression.

Conversely, in ovarian cancer, DDR1 sustains collagen-induced cisplatin resistance independently of intβ1, acting as an alternative collagen sensor for mediating matrix-induced resistance [87]. Differently, in breast cancer, intβ1 and DDR1 converge on MAPK signaling underpinning a highly dynamic resistance mechanism. Intβ1 promotes chemoresistance via ATP-Binding Cassette efflux transporters [88], while DDR1 acts as a backup collagen sensor when intβ1 is absent, maintaining MAPK activation in response to collagen [89].

DDRs and integrins differently regulate cancer cell fate, influencing dormancy, reactivation, and survival through distinct mechanisms. For instance, intα3β1 drives the reactivation of dormant cancer cells in a mouse lung metastasis model. Persistent lung inflammation promotes the formation of neutrophil extracellular traps (NETs), which proteolytically remodel laminin to expose a cryptic epitope activating integrin signaling, reawakening dormant cancer cells and promoting metastatic outgrowth [90]. In contrast, DDR2 regulates neuroblastoma cell fate by translating mechanical cues into transcriptomic reprogramming, modulating survival, and differentiation pathways [91]. Meanwhile, DDR1 has been implicated in tumor cell dormancy across various experimental models by upregulating type III collagen via STAT1 activation [92].

In some cases, the strength of mechanical signals determines distinct cellular outcomes driven by DDRs and intβ1 role in cell fate processes. In breast tumor cells, Li et al. demonstrated that DDR2 promotes tumor cell quiescence by inducing cell cycle arrest via the STAT1/p27 pathway, whereas intβ1/β3 enhances stem-like traits and tumorigenic potential receptors under low ECM stiffness [93]. This study underscores how DDR2 and integrins differentially transduce mechanical cues from ECM, ultimately shaping breast cancer cell behavior and proliferative capacity. Similarly, in rhabdomyosarcoma, stiff collagen activates intβ1 to support cell survival, while low remodeling induces apoptosis [94].

These findings highlight DDRs and integrins as context-dependent therapeutic targets. While DDR inhibition may overcome ECM-mediated drug resistance in melanoma, their role in dormancy complicates broad inhibition strategies. Conversely, targeting integrin-mediated mechanosignaling could disrupt metastasis-prone niches. Further studies should explore combinatorial approaches to selectively disrupt pro-tumoral ECM signaling.

A unique cooperative mechanism between intβ1 and DDR1 has been found in the context of pancreatic ductal adenocarcinoma (PDAC). Pancreatic cancer cells produce a distinct Col1 homotrimer that binds intα3β1, establishing a persistent proliferation signal axis. This collagen-integrin interaction simultaneously activates DDR1, creating a dual receptor signaling network. Notably, while intβ1 knockdown abolishes collagen homotrimer-induced signaling, DDR1 suppression leads to paradoxical pathway hyperactivation, suggesting compensatory signaling mechanisms. This finding reveals the complex interplay between these two collagen receptors in PDAC pathogenesis. Noteworthy, Col1 deletion in cancer cells enhances T cell infiltration and enables efficacy of anti-PD-1 immunotherapy [95].

This highlights the collagen-rich PDAC microenvironment as both a mechanical barrier and an immune-suppressive factor, with DDR1-integrin cooperation playing a central role.

### Intβ1 and DDRs crosstalk in collagen-driven ECM remodeling

Collagen synthesis is primarily regulated at the transcriptional level, a process influenced by external cues, such as mechanical signals and ECM composition. Both integrins and DDRs sense collagens and activate shared downstream signaling pathways (e.g., MAPK, Src, PI3K/Akt), which modulate fibroblast behavior and collagen gene expression. While no direct evidence has been reported supporting cooperation among DDR and integrins in collagen synthesis, both receptors coordinate collagen sensing and response, indirectly influencing collagen remodeling and deposition (Table 2).

**Table 2:** Role of DDR and intβ1 in collagen deposition and remodeling

Experimental model	Key findings	ECM target	Citations
Human fibroblasts	DDR2 sustains MT1-MMP independently of intβ1	MT1-MMP	[102]
Skin fibroblasts	DDR2 sustains MMP2 expression and activity	MMP2	[103]
Cardiac fibroblasts	Ang II-stimulated DDR2 regulates intβ1 expression, which in turn modulates α-SMA and collagen I expression	Collagen I	[79]
Hepatic stellate cells	DDR2 regulates MMP2 expression	MMP2	[104]
GD25 embryonic stem cells	DDR1 in complex with MVP inhibits intβ1-mediated MMP1 activation	MMP1	[105]
Breast cancer cells	DDR2 sustains MT1-MMP independently of intβ1	MT1-MMP	[106]

List of evidence linking DDRs and intβ1 in the context of collagen-driven ECM remodeling

DDRs has been linked to upregulation of Col1 expression in fibroblasts and fibrosis, as well as to matrix remodeling modulating collagen-modifying enzymes, like MMPs or LOX [96–99]. On the other hand, integrins contribute to collagen synthesis or modulate TGF-β activation, a strong inducer of collagen synthesis [100, 101].

In human fibroblasts, DDR2, but not integrins, mediates collagen-induced activation of Membrane-Type1 (MT1)-MMP. While inhibition of DDR or Src kinases suppresses MT1-MMP activation, intβ1 knockdown had no effect [102]. Besides MT1-MMP, DDR2 promotes MMP-2 expression and activity by transcriptionally activating the MMP-2 promoter in skin fibroblasts. The lack of physical association between DDR2 and intβ1 suggests a DDR2 dominance in MMP-2 expression regulation, although not excluding the convergence of DDR2 signals with those mediated by integrins downstream of collagen binding [103]. A prominent role of DDR2 in the regulation of MMP2 has also been demonstrated in hepatic stellate cells, where intβ1 involvement had been previously excluded [104].

Different from the above-described works, a regulatory feedback mechanism between DDR1 and intβ1 signaling pathways has been demonstrated in regulating collagen remodeling [105]. Specifically, activated DDR1 binds the scaffolding protein major vault protein (MVP), uncoupling intβ1 from the FAK/ERK pathway and suppressing MMP1 expression. Thus, DDR1 dampens intβ1-dependent collagen degradation via interaction with MVP [105].

In breast cancer, activated DDR2 stabilizes SNAIL1, sustaining MT1-MMP production and activity and collagen synthesis, both of which contribute to the remodeling of collagen fibers at the tumour/ECM interface. This creates a feedforward loop driving breast cancer invasion and migration in vitro and metastasis in vivo. Notably, Col1-induced SNAIL1 stabilization occurs independently of integrin and TGFβ, highlighting the unique function of DDR2 in sustaining EMT phenotype and tumor cell invasion through Col1-rich ECM. The fact that DDR2 depletion reduced in vivo metastases imply not redundant role during breast cancer metastasis [106].

Interestingly, a unique spatial localization of DDR2 and intβ1 collagen binding domain has been reported by Macdonald et al. They mapped collagen-binding domains by spatial proteomic approaches in hepatocellular carcinoma in two subtypes characterized by opposite outcomes. Fibrotic S1 subtype displays strong TGFβ1-driven collagen deposition associated with poor outcome, while S3 subtype with WNT pathway activation associates with a better outcome [107]. They identified two key functional domains, DDR2-binding motif (GVMGFP), critical for collagen-DDR2 interactions, and intαβ binding (motif GFPGER), mediating collagen-integrins binding. Neither DDR2- nor integrin-binding domains distinguish S1 from S3 subtype at the bulk level. However, they found a distinct localization pattern. DDR2-binding peptide distributed across tumor tissue and bridged fibrosis in both subtypes, suggesting a broad role in collagen sensing. The integrin-binding peptide showed intense, focal expression within S1 tumor regions, aligning with their fibrotic phenotype and peritumoral enrichment in S3 subtypes [107]. This unique spatial localization with complementary patterns across the tissues suggests a complementary role of the two receptors in stroma crosstalk deposition.

### Therapeutic combinational settings: a potentially promising approach

Integrins have long been recognized as both promising and complex targets for treating a wide range of diseases, including cancer, fibrotic conditions, cardiovascular disorders, viral infections, and autoimmune diseases. Despite ongoing advances in understanding the role of integrins in various pathologies, the progress in drug development has been relatively unsatisfactory. Currently, there are approximately 90 integrin-targeting therapies undergoing clinical trials, which include both monoclonal Abs (mAbs) and small-molecule drugs [108]. Importantly, current research is expanding beyond extracellular targets to also explore intracellular mechanisms, addressing both inside-out and outside-in integrin signaling pathways.

On the other hand, although significant progress has been made in understanding the roles of DDRs in pathological contexts, including cancer, critical knowledge gaps and underexplored areas remain, hindering the creation of fully effective therapeutic strategies. Addressing these gaps will accelerate the development of targeted treatments, especially in cancer, where tumor heterogeneity, the evolving TME, and individual patient differences remain the major critical issues. Currently, most DDR-targeted therapies emphasize kinase inhibition [109]. However, increasing evidence highlight the significance of non-kinase functions of DDRs in the development of cancer, with emerging approaches capable of targeting their expression and functionality. Recent research has indicated the potential for the utilization of DDR degraders. In a model of NSCLC, Xue and colleagues [110] identified NSC632839, a small-molecule compound capable of inducing DDR1 degradation by impairing USP7, a novel deubiquitylating enzyme (DUB). Considering that USP7 inhibition leads to the restoration of p53 expression, this approach could be a viable means of targeting DDR1 in tumors arising from p53 mutations. In this particular context, the development of proteolysis-targeting chimeras (PROTACs) has given rise to a novel therapeutic approach, which involves the degradation of target proteins through the ubiquitin–proteasome system [111]. DP1, a novel PROTAC-based DDR1 degrader, has been evaluated as a drug with both high selectivity and potent degradation efficacy across a range of cancer cell lines. In vivo, it has been demonstrated to both inhibit DDR1 signaling and disrupt the immune barrier [111]. The drug demonstrates excellent potential in terms of draggability and biosafety [112]. It is important to note that no data are available for DDR2. Regarding integrins, few data are available in which a PROTAC targeting PD-1/PD-L1 was designed using the tumor-penetrating iRGD peptide, binding intαvβ3 and inducing neuropilin-1-mediated transcytosis for deep tumor penetration but not considering integrins as therapeutic targets [113, 114]. A recent study conducted on melanoma cells has demonstrated that DUBs function as sensors for mechanical signaling from the ECM, requiring DDRs for the adaptation of tumor cells to high stiffness [115]. A screening strategy based on melanoma cells cultivated on collagen matrices with various stiffnesses, combined with an activity-based ubiquitin probe for profiling DUB activity and quantitative proteomics, identified USP9X, a highly conserved DUB, as capable to prevent YAP degradation through the DDR/actomyosin signaling pathway [115]. Considering the established position of YAP at the nexus of integrin and DDR signaling, further research is imperative to ascertain the significance of this mechanism in the context of integrin signaling and the potential interaction with DDR signaling. The promising outcomes of various DDR inhibitors in recent studies suggest an expanding scope for future research. As the roles of DDRs in different types of cancer continue to be uncovered, novel functions are expected to emerge, guiding the design of more precise and personalized therapeutic approaches, considering the interplay with integrins.

Targeting the ECM has emerged as a promising therapeutic strategy in combination with collagen receptors. Recent studies underscored the efficacy of combined therapy by using integrin inhibitors with MMP inhibitors in cancer [116]. Most importantly, recent advances revealed that targeting DDRs reinforces the response to immunotherapy in cancer, reflecting the restored "hot" inflammatory TME [50]. In this context, the efficacy of an anti-DDR1 antibody to the DDR1 extracellular domain in collagen fiber rearrangement, enhancing T cell infiltration [117], led to the development of DDR1-DX8951, an antibody–drug conjugate that includes an anti-DDR1 mAb in combination with the immune checkpoint inhibitor, pembrolizumab, which revealed to be efficacy in the inhibition tumor growth and metastasis and in improving the immunosuppressive TME in preclinical models of TNBC [118]. To overcome both physical and immune barriers in order to reach the core of the tumor and reconfigure TME, an inhalable lipid nanoparticle (LNP)-mediated RNA therapeutic approach combining mRNA encoding anti-DDR1 single-chain variable fragments and siRNA targeting PD-L1 has been tested in both orthotopic and metastatic mouse models of lung cancer [119]. With this approach, the infiltration of immune effector cells, including T cells, NK cells, and NKT cells, while reducing the presence of immunosuppressive cells in the TME, results in restrained tumor growth and prolonged overall survival, further confirming the importance in the targeted therapy to alter the collagen alignment pattern in order to reduce tumor stiffness and obtaining a TME more conducive to immune cell infiltration. From a translation point of view, clinical trials using DDR1 humanized mAbs in advanced solid tumor patients are currently ongoing

(https://clinicaltrials.gov/study/NCT05753722) [120]. Regarding DDR2, advances in nanomedicine by introducing a biomimetic stroma-modulating platform that combines co-deliver of DDR2-based CAF targeting with WRG-28 and a photothermal agent (IR-780), showed the efficacy of this dual-action strategy to suppress metastasis, induced CAF apoptosis, and dismantled the fibrotic barrier while preventing CAF repopulation in murine breast cancer models [121].

Similar effects have been demonstrated in experimental glioma models, where an integrin-blocking peptide prevents the reprogramming of immunosuppressive myeloid cells, resulting in the conversion of a “cold” to a “hot” TME, boosting innate and adaptive immune responses elicited by anti-PD-1 Abs and normalizing the tumor vasculature, leading to eradication of the tumors, or glioblastoma were an antibody blocking intα5 enhanced anti-PD-1-mediated antitumor immunity by remodeling tumor-associated macrophages [122, 123].

While novel strategies targeting integrins and DDRs have shown promise in preclinical models, the therapeutic co-targeting remains elusive. This might be attributable to a poor understanding of the intricate, cell-type specific molecular dialogue between these pathways.

The findings discussed above underscore that, besides functioning as independent sensors of collagen, DDRs and integrins can engage in context-dependent, finely tuned crosstalk, generating overlapping, synergistic or competing signaling pathways, or microenvironment modulation, as observed with stromal cells and fibrosis, having a crucial role in ECM-related diseases. Therefore, a therapeutic combination targeting both integrins and DDRs is a scientifically plausible and potentially promising approach. In the context of cancer, therapy combining DDRs and intβ1 targeting could more effectively reprogram the TME to make it less supportive of cancer progression.

Emerging advances in regenerative medicine and oncology underscore the therapeutic potential of concomitant targeting both DDR2 and intβ1. While these receptors independently regulate bone development and responsiveness of bone morphogenetic proteins to mechanical stimuli from ECM [42, 48], their functional cooperation comes from Franceschi group’s studies [124]. Using triple-helical peptides, they demonstrated that activation of DDR2 and integrins synergistically enhances osteoblast differentiation. Interestingly, DDR2 was required for full integrin signaling, revealing cross-dependence [124]. Building on this, Zhou et al. designed peptide-conjugated nanofibrous polymer scaffolds combining DDR2- and integrin-activating triple-helical peptides, which were more effective than single-peptide systems at promoting bone regeneration in osteogenesis [125].

In cancer, dual blockade of DDR2 and Intαv subfamily (ITGAV) via siRNA-loaded nanocarriers has been pursued as a strategy to overcome matrix barriers to enhance immune infiltration in preclinical models of breast cancer. This dual approach effectively decreased collagen deposition, increased CD8 + T cell infiltration, and suppressed PD-L1 expression in murine models of triple-negative breast cancer, surpassing that of targeting either receptor alone, highlighting a synergistic ECM reprogramming deriving from DDR2 ability to mediate collagen-cell interactions and ITGAV ability to sustain TGF-β1 activation. These findings position DDR2/integrin co-targeting as a value therapeutic paradigm for cancer immunotherapy, with potential applications in triple-negative breast cancer as well as other collagen-rich solid tumors. This approach may also synergize with other existing therapies for tumors that can benefit from overcoming immune exclusion.

Similarly, in glioblastoma combined inhibition of DDR1 and intαVβ3/αVβ5 using DDR1-IN-1 and cilengitide impaired DNA double-strand break (DSB) repair by altering Ataxia-Telangiectasia Mutated protein kinase (ATM) and DNA-dependent protein kinase (DNA-PK) phosphorylation, radiosensitized tumors and correlated with worse patient survival [126]. While more research is needed, especially in vivo models and clinical settings, this approach has the potential to yield synergistic anti-tumor effects and overcome resistance mechanisms.

## Conclusions

The intricate and context-dependent interplay between integrins and DDRs represents a master regulatory mechanism by which cells decode the biochemical and biomechanical language of the collagenous ECM. This review synthesizes compelling evidence that these receptors do not operate in isolation; their crosstalk, ranging from synergistic and compensatory to antagonistic, is a central processing unit for collagen-derived signals, whose output is fundamental to physiological processes like development and tissue repair;  its dysregulation dictates cell fate in fibrosis, cancer, and aging. The critical unanswered question is no longer if they crosstalk, but *how, when,* and *where* this occurs to produce specific cellular outcomes. Future efforts must focus on spatiotemporal mapping of this interplay by employing advanced imaging and spatial proteomics to visualize the dynamic localization and segregation of DDR and integrin in real-time within living tissues and mechanistic decoding, with the aim to identify the exact molecular intermediaries that facilitate the conformational activation of integrins by DDRs and vice versa.

The functional redundancy and crosstalk between these receptors explain the limited success of monotherapies. Preclinical models demonstrate that dual targeting synergistically reprograms the ECM, enhances immune infiltration, and overcomes drug resistance highlighting the unmet need to designing smart, context-sensitive combinatorial strategies, such as bispecific Abs, nanocarriers, or selective inhibitors, that target tumor-specific receptor complexes or ECM post-translational modifications, thereby minimizing on-target and off-site toxicity. By answering these calls to action, we can move from simply inhibiting these receptors to therapeutically reprogramming their communication, ultimately dismantling the pathological ECM that fuels fibrosis and cancer, restoring tissue homeostasis. Advancements in bioinformatic tools combined with artificial intelligence-driven methodologies show promise to identify novel inhibitors tailored exclusively towards DDRs.

## Data Availability

No datasets were generated or analyzed during the current study.

## Abbreviations

AGE: Advanced glycation end-product
Ang II: Angiotensin II
BRAF: Proto-oncogene B-Raf
CAF: Cancer-associated fibroblast
DDR: Discoidin domain receptor
DUB: Deubiquitylating enzyme
DS: Discoidin homology domain
ECM: Extracellular matrix
EMT: Epithelial-mesenchymal transition
ERK1/2: Extracellular signal-regulated kinase 1/2
FAK: Focal adhesion kinase
GFO: Glycine–phenylalanine–hydroxyproline
IAC: Integrin adhesion complex
Intβ1: Integrin β1
ITGAV: Integrin α V subfamily
LOX: Lysyl oxidase
MAPK/ERK: Mitogen-activated protein kinase/extracellular-signal-regulated kinase
MMP: Matrix metalloproteinase
MT1-MMP: Membrane-type 1-MMP
MVP: Major vault protein
NF-κB2/RelB: Nuclear factor kappa-light-chain-enhancer of activated B cells 2 protein
NMIIA: Non-muscle myosin IIA
PD-1: Programmed death-1
PDAC: Pancreatic ductal adenocarcinoma
PD-L1: Programmed death-ligand 1
PI3K/Akt: Phosphatidylinositol 3-kinase/Protein Kinase B
Shc: SHC-transforming protein 1
SHP-2: Src homology 2 domain-containing protein tyrosine phosphatase-2
TGF-β1: Transforming growth factor-1
TME: Tumor microenvironment
TKR: Tyrosine kinase receptor
TVC: Trunk ventral cell
WNT: Wingless-related integration site
