# Correction: Decoding collagen cues: the interplay of integrins and discoidin domain receptors in health and disease

**DOI:** 10.1186/s12929-026-01235-0

**Published:** 2026-03-19

**Authors:** Paola Trono, Ilenia Masi, Flavia Ottavi, Laura Rosano

**Affiliations:** 1 Institute of Biochemistry and Cell Biology (IBBC)-CNR, Via E. Ramarini, 32, 00015 Monterotondo Scalo, Rome, Italy; 2 Institute of Molecular Biology and Pathology (IBPM)-CNR, Via Degli Apuli 4, 00185 Rome, Italy

**Correction: Journal of Biomedical Science (2026) 33:8** 10.1186/s12929-025-01211-0

After publication of the article [1], it was brought to our attention that the first name and last name of author Flavia Ottavi are reversed. The correct name has been shown in this Correction.

The original article has been updated.
